# Adolescent Stress Reduces Adult Morphine-Induced Behavioral Sensitization in C57BL/6J Mice

**DOI:** 10.3389/fnbeh.2021.678102

**Published:** 2021-06-03

**Authors:** Helen M. Kamens, Carley N. Miller, Jasmine I. Caulfield, Dana Zeid, William J. Horton, Constanza P. Silva, Aswathy Sebastian, Istvan Albert, Thomas J. Gould, Diana Fishbein, Patricia Sue Grigson, Sonia A. Cavigelli

**Affiliations:** ^1^Department of Biobehavioral Health, The Pennsylvania State University, University Park, PA, United States; ^2^The Huck Institutes of the Life Sciences, The Pennsylvania State University, University Park, PA, United States; ^3^Department of Psychology, Bucknell University, Lewisburg, PA, United States; ^4^Biochemistry and Molecular Biology, The Pennsylvania State University, University Park, PA, United States; ^5^Department of Human Development and Family Studies, The Pennsylvania State University, University Park, PA, United States; ^6^FPG Child Development Institute, The University of North Carolina at Chapel Hill, Chapel Hill, NC, United States; ^7^Department of Neural and Behavioral Sciences, The Pennsylvania State University, Hershey, PA, United States

**Keywords:** morphine, social stress, adolescence, consumption, sensitization, miRNA, prefrontal cortex

## Abstract

Deaths related to opioid use have skyrocketed in the United States, leading to a public health epidemic. Research has shown that both biological (genes) and environmental (stress) precursors are linked to opioid use. In particular, stress during adolescence–a critical period of frontal lobe development–influences the likelihood of abusing drugs. However, little is known about the biological mechanisms through which adolescent stress leads to long-term risk of opioid use, or whether genetic background moderates this response. Male and female C57BL/6J and BALB/cJ mice were exposed to chronic variable social stress (CVSS) or control conditions throughout adolescence and then tested for morphine locomotor sensitization or morphine consumption in adulthood. To examine possible mechanisms that underlie stress-induced changes in morphine behaviors, we assessed physiological changes in response to acute stress exposure and prefrontal cortex (PFC) miRNA gene expression. Adolescent stress did not influence morphine sensitization or consumption in BALB/cJ animals, and there was limited evidence of stress effects in female C57BL/6J mice. In contrast, male C57BL/6J mice exposed to adolescent CVSS had blunted morphine sensitization compared to control animals; no differences were observed in the acute locomotor response to morphine administration or morphine consumption. Physiologically, C57BL/6J mice exposed to CVSS had an attenuated corticosterone recovery following an acute stressor and downregulation of twelve miRNA in the PFC compared to control mice. The specificity of the effects for C57BL/6J vs. BALB/cJ mice provides evidence of a gene-environment interaction influencing opioid behaviors. However, this conclusion is dampened by limited locomotor sensitization observed in BALB/cJ mice. It remains possible that results may differ to other doses of morphine or other behavioral responses. Long-term differences in stress reactivity or miRNA expression in C57BL/6J mice suggests two possible biological mechanisms to evaluate in future research.

## Introduction

The use of opioids is a widespread public health crisis. Between 1999 and 2018 opioid overdoses accounted for 440,000 deaths in the United States (Wilson et al., 2020). This number has been on the rise with over 46,000 Americans losing their lives to an opioid overdose in 2018 alone (Wilson et al., 2020). These deaths arise from the 10.3 million individuals who report misusing opioids each year (Substance Abuse and Mental Health Services Administration, 2019). Given the prevalence of opioid abuse in the United States, research into the mechanisms that alter the risk of opioid use is important for combating this public health crisis.

Opioid abuse is influenced by multiple factors. Human genetics studies have demonstrated that genes influence opioid use and dependence with heritability estimates ranging from 0.52 to 0.76 (van den Bree et al., 1998; Kendler et al., 1999, 2000; Sun et al., 2012). In this work, the use of different types of opioids (e.g., morphine, oxycodone, and fentanyl) is not differentiated. However, research with inbred panels of mice have confirmed a genetic influence for individual opioids, including morphine, with heritability estimates of morphine behavioral responses ranging from 0.26 to 0.44 (Bergeson et al., 2001; Kest et al., 2002; Liang et al., 2006). Although these findings support genetic influences on opioid use and responses, environmental factors also impact opioid use.

One environmental influence linked to opioid use is exposure to stress. Research in animal models has demonstrated that exposure to social stressors can influence opioid behaviors (Neisewander et al., 2012). Limiting results to only mouse models provides a more consistent pattern. In male C57BL/6J mice, exposure to chronic social stress in adulthood increased morphine preference in a 2-bottle choice experiment immediately after the stressor, but the effect was not long lasting (Cooper et al., 2017). Also, exposure to social defeat in adult male OF1 mice reinstated morphine conditioned place preference following extinction (Ribeiro Do Couto et al., 2006). These data suggest that adult social stress can influence morphine behaviors; however, there is limited data on how social stress during adolescence influences behavior related to drug use. To our knowledge there has been one study that examined the effect of an adolescent stressor, social isolation for 30 days, on the rewarding effects of morphine in mice. In male NMRI, morphine conditioned place preference was eliminated in animals raised in isolation (Coudereau et al., 1997). Determining effects of stress on opioid behaviors in both sexes is critical as prior research has demonstrated that the risk factors that lead to opioid misuse may differ between men and women. Specifically, women reported heightened distress as a risk factor for opioid abuse where as men reported greater legal or behavioral problems (Jamison et al., 2010). The pre-clinical literature described above was conducted in male mice demonstrating a need to determine if these effects also occur in female animals.

Adolescence is a critical time of brain and social development (Spear, 2013) and stress during this time generally leads to increased substance use in both humans and in animal models (Dube et al., 2003; Andersen and Teicher, 2009). In rodents, adolescence is generally defined as approximately postnatal days (PND) 28–60 when they show signs of behavioral, neurobiological, and pubertal changes that are akin to human adolescence (McCormick and Mathews, 2007). The prefrontal cortex (PFC), which is responsible for executive functions, such as decision making, impulse control, and working memory has important connections with limbic structures involved in drug use. This brain region undergoes numerous structural and functional changes during adolescence. These dynamic changes open a window for environmental factors, particularly stress, to modulate processes associated with drug use (Jankord et al., 2011). In particular, the PFC is involved in regulating the hypothalamic-pituitary-adrenal (HPA) axis which matures during adolescence and has been implicated as one pathway by which social stress may affect opioid behavioral responses in adult rats (Deroche et al., 1994, 1995). One long-lasting molecular change found as a result of adolescent stress is miRNA expression patterns (Rao and Pak, 2016; Liu et al., 2017; Xu et al., 2017). These small RNA molecules regulate the expression of messenger RNA molecules, and thus could lead to wide scale changes in gene expression profiles, neuronal function, and altered drug behaviors (Smith and Kenny, 2018).

The current study aims to investigate both environmental and genetic influences on opioid behaviors in a mouse model. We examined the influence of adolescent social stress on opioid behaviors in two different inbred mouse strains. The C57BL/6J and BALB/cJ strains were chosen because they are sensitive to long-term alterations in drug behaviors following chronic social stress in adolescence (Caruso et al., 2018a,b,c). In prior research, adult social stress (isolation or social defeat) led to increased voluntary morphine consumption (Alexander et al., 1978; Raz and Berger, 2010; Cooper et al., 2017). Thus, we hypothesized that chronic variable social stress (CVSS), a model of adolescent social stress that includes repeated cycles of social isolation and social re-organization, would increase morphine consumption. The direction of effect we expect to observed with morphine behavioral sensitization is less clear. It is possible we will observe increased sensitization for similar reasons that we hypothesize increased morphine consumption. However, data in pre-clinical models has demonstrated that adolescent stress diminishes morphine conditioned place preference (Wongwitdecha and Marsden, 1996; Coudereau et al., 1997), so it is equally reasonable to hypothesize that we will observe diminished sensitization following adolescent stress. To identify a potential mechanism by which adolescent stress alters morphine behaviors, we measured corticosterone (CORT) responses to an acute stressor and miRNA expression patterns in the PFC. As above, this brain region was chosen because it is known to be susceptible to stress manipulations and to influence drug behaviors (Andersen and Teicher, 2009). Further, in prior work we have shown long-term alterations in PFC excitatory transmission following CVSS (Caruso et al., 2018a).

## Materials and Methods

### Animals

Male and female BALB/cJ and C57BL/6J mice (The Jackson Laboratory, Bar Harbor, ME, United States) were bred at The Pennsylvania State University. On PND 21 offspring were weaned into groups of 2–3 same sex littermates housed in polycarbonate cages (28 cm × 17 cm × 12 cm) lined with corn-cob bedding. The animals were maintained on a 12-h light-dark schedule (lights on at 0700 h) in a temperature- and humidity-controlled facility with *ad libitum* food and water available. All procedures were approved by The Pennsylvania State University IACUC committee.

### Drugs and Solutions

For morphine consumption, morphine sulfate pentahydrate was obtained from the NIDA Drug Supply Program (Bethesda, MD, United States), while saccharin sodium salt and quinine hemisulfate salt were obtained from Sigma-Aldrich (St. Louis, MO, United States). Morphine, saccharin, and quinine were diluted in tap water for the consumption study (see below). For locomotor sensitization, morphine sulfate pentahydrate (Sigma-Aldrich, St. Louis, MO, United States) was diluted in physiological saline (0.9% NaCl; Baxter) and injected intraperitoneally in a 10 ml/kg volume. The two suppliers of morphine were based on differences in funding sources between projects.

### Chronic Variable Social Stress (CVSS)

To minimize litter effects, littermates were evenly distributed between the CVSS and control groups at weaning. CVSS mice underwent the stress protocol during adolescence (PND 25–59) as previously described (Caruso et al., 2017, 2018a,b,c). Following weaning mice sat undisturbed with littermates (PND 21–25). On PND 25, CVSS mice were individually housed for 3 days followed by social rehousing with 1–2 unfamiliar cage mates for 4 days. This procedure was repeated for five consecutive weeks. On PND 59, CVSS mice were rehoused with their original cage mates from weaning until behavioral testing or sacrifice. For control mice, cage mates remained the same from weaning to the end of the study. To equate handling between the control and CVSS conditions, each time CVSS mice were transferred to a new cage for individual or social rehousing, control mice were also moved to new cages. Table 1 details the number of CVSS and control animals tested for each behavioral outcome described in detail below. Importantly, all behavioral and physiological outcomes were examined in independent groups of animals.

**TABLE 1:** Overview of experimental design.

Study	Subjects	Outcome
1	C57BL/6J CON = 12 M & 15 F	Morphine Sensitization
	C57BL/6J CVSS = 14 M & 11 F	
	BALB/cJ CON = 11 M & 14 F	
	BALB/cJ CVSS = 11 M & 14 F	

2	C57BL/6J CON = 11 M & 11 F	Morphine Consumption
	C57BL/6J CVSS = 15 M & 12 F	
	BALB/cJ CON = 15 M & 11 F	
	BALB/cJ CVSS = 14 M & 14 F	

3	C57BL/6J CON = 9 M & 5 F	Acute Stress CORT Response
	C57BL/6J CVSS = 8 M & 6 F	

4	C57BL/6J CON = 6 M	Prefrontal Cortex miRNA
	C57BL/6J CVSS = 5 M	

*CON = control; CVSS = chronic variable social stress; M = male; F = female; CORT = corticosterone.*

### Methods of Behavioral and Physiological Outcomes

#### Morphine Sensitization

Approximately 1 week after CVSS was complete, adult mice (PND 65–72) were tested for morphine-induced locomotor sensitization using published methods (Phillips et al., 1994b; Kamens et al., 2005). To achieve the desired sample size, two sequential cohorts were used that were counterbalanced for strain, sex, and stress condition. This age was chosen because our prior work demonstrated changes in drug behavior and physiological responses at this time (Caruso et al., 2017, 2018a,b,c). Testing was conducted between 0900 and 1300 h. On test days, all animals were moved to the experimental room and allowed to acclimate for at least 30 min. To acclimate mice to the test chambers, on days 1–2 all mice received an acute injection of saline immediately prior to being placed into a Superflex locomotor activity chambers (Omnitech Electronics, Inc., Columbus, OH, United States). On day 3, animals were randomly assigned to the chronic saline or chronic drug group. Chronic saline mice received saline and chronic drug mice received 100 mg/kg morphine on days 3, 5, 7, and 9 immediately before being placed in the activity chamber. On day 11, mice in both the chronic saline and chronic drug conditions received 100 mg/kg morphine (Table 2 depicts full protocol). This high dose of morphine was chosen because it has been shown to elicit the development of behavioral sensitization in adolescent and adult C57BL/6J animals (Koek, 2014) and was used in prior work on long term effects of adolescent stressors in male NMRI mice (Coudereau et al., 1997). On each day, locomotor activity was measured for 30 min and data were recorded as centimeters (cm) traveled. Three scores were calculated to examine acute morphine locomotor stimulation and sensitization within the chronic saline or chronic drug groups. Acute morphine stimulation was calculated as Day 3–Day 2 locomotion in the chronic drug group and Day 11–Day 2 locomotion in the chronic saline group. Sensitization in the chronic drug group was measured as Day 11–Day 3 locomotion (Kamens et al., 2005). In each strain, 12–14 mice were tested in each of the four stress × drug groups (Control-Chronic Drug, Control-Chronic Saline, CVSS-Chronic Drug, CVSS-Chronic Saline), with approximately equal numbers of males and females in each group.

**TABLE 2:** Treatment protocol for morphine sensitization protocol.

Group	Day 1	Day 2	Day 3	Day 4	Day 5	Day 6	Day 7	Day 8	Day 9	Day 10	Day 11
***Chronic Saline***											
Injection	Saline	Saline	Saline	None	Saline	None	Saline	None	Saline	None	MO
Test	Yes	Yes	Yes	No	Yes	No	Yes	No	Yes	No	Yes
***Chronic Drug***											
Injection	Saline	Saline	MO	None	MO	None	MO	None	MO	None	MO
Test	Yes	Yes	Yes	No	Yes	No	Yes	No	Yes	No	Yes

*MO: Morphine.*

#### Morphine Consumption

With a separate cohort of adult mice (starting PND 65–68) 2-bottle choice morphine consumption was tested using published methods (Eastwood and Phillips, 2014). Mice were individually housed and provided with two 25-ml graduate cylinders fitted with sipper tops. To acclimate mice to the new test environment, water was available for 2 days in both tubes. After acclimation, water tubes were replaced with one tube containing 0.2% saccharin plus morphine while the other tube contained 0.2% saccharin plus quinine. Quinine was provided as the alternative solution to account for the bitter taste of morphine following published protocols (Eastwood and Phillips, 2014). Drug solutions were available 24 h a day. Morphine/quinine concentration increased every 4 days (morphine: 0.3, 0.7, and 1 mg/ml; quinine: 0.2, 0.4, and 0.55 mg/ml). Body weight was measured each time the drug concentration changed. The side of the cage where morphine was presented was alternated every other day to prevent the development of a side preference. Morphine consumption (mg/kg), quinine consumption (mg/kg), and total fluid consumption (ml) were calculated and used as dependent variables. For each drug concentration, the average of day 2 and 4 consumption values were used to assess stable drug intake (Phillips et al., 1994a). For each strain, 22–28 mice were tested from each stress condition, evenly split between males and females.

#### Acute Stress

Our data indicated that C57BL/6J, but not BALB/cJ mice, showed different behavioral responses to morphine following adolescent stress (see section “Results”), therefore the remaining measures were limited to this strain. Adult C57BL/6J mice (PND 81–83) were used to measure the glucocorticoid response to an acute stressor. We used a standard restraint stress protocol administered between 0900 and 1100 h. Mice were removed from their home cage and placed in a broom-style restrainer. The tip of the tail (<1 mm) was removed and a baseline blood sample was collected within 3 min of initial disruption. Mice remained in the restrainer for 15 min then were placed into individual holding cages. At 30 and 90 min after initial placement in the restraint tube, mice were briefly returned to restrainers for an additional blood sample collection. These time points were chosen to capture peak (30 min) and recovery (90 min) CORT levels following an acute stressor (Romeo, 2010). Stress reactivity was defined as 30 min CORT–baseline CORT and recovery was defined as 90 min CORT–30 min CORT (Sapolsky et al., 1983). These two scores were used as dependent variables. Fourteen CVSS and 14 control animals were tested in this protocol including both sexes. Blood samples were collected into heparinized capillary tubes and immediately stored on ice. Plasma was obtained by centrifuging samples for 15 min at 10,000 × *g* at 4°C then stored at −80°C. CORT was measured using a commercial [^125^I] radioimmunoassay kit (MP Biomedicals, Solon, OH, United States) following manufacturer guidelines (Caruso et al., 2018a,b). Intra- and inter-assay coefficients of variation were 7.8 and 0.8 (for low control) and 7.5 and 7.5 (for high control).

#### Prefrontal Cortex (PFC) miRNA Gene Expression

To examine adolescent stress-induced alterations in miRNA gene expression 11 (6 control, 5 CVSS) male C57BL/6J mice, unexposed to opioids, were used. Only C57BL/6J males were examined because they exhibited a change in morphine sensitization following CVSS (see section “Results”). Following CVSS, these mice remained undisturbed (i.e., no behavioral testing) until PND 70 when they were euthanized by cervical dislocation and brains removed. A mouse brain matrix was used to obtain 1 mm slices. The PFC was punched from the slice with a blunt needle that captured both hemispheres and immediately place into RNAlater. Tissue was homogenized and RNA was extracted using the RNeasy^®^ Plus Universal Mini Kit (Qiagen, Valencia, CA, United States) following the manufacturers’ guidelines for the isolation of total RNA including small RNAs. RNA quality was determined using the RNA Integrity Number (RIN) with an Agilent 2100 Bioanalyzer (Santa Clara, CA, United States). The average RIN of our samples was 9.2 (range 8.9–9.3), suggesting high quality (Schroeder et al., 2006).

RNA-Seq libraries were prepared using the TruSeq Small RNA Library Construction Kit (Illumina, San Diego, CA, United States). The resulting libraries were quantified and pooled for sequencing on an Illumina HiSeq to obtain 50 bp reads. On average we obtained 7 million reads per sample with and average mapping rate of 81%. Library preparation and sequencing was conducted in the Penn State Genomics Core Facility, University Park, PA. Sequencing data are available from the NCBI GEO database (experimental series accession number: GSE172077).

Sequencing reads were mapped to the mouse genome (*Mus musculus* mm10) using ShortStack (Johnson et al., 2016). miRNA annotations were downloaded from miRBase and reads mapped to miRNAs were counted in ShortStack using the default settings. Differential expression between groups was performed using DESeq2 (Love et al., 2014). *P*-values were corrected for multiple comparisons using a Benjamini-Hochberg false discovery rate of 5% (Benjamini and Hochberg, 1995).

Differentially expressed miRNAs were analyzed for predicted mRNA targets and associated biological pathways using the DIANA toolkit^1^ and Ingenuity Pathway Analysis (IPA) software (content version 52912811; Krämer et al., 2014). The number of miRNA-gene relationships was predicted using the DIANA microT-coding sequence (CDS) tool, v.5 (Paraskevopoulou et al., 2013), which computes miRNA-mRNA interaction scores for predicted targets in mRNA 3′-UTR and CDS transcript regions. The microT-CDS score threshold was set to the default 0.8, which is recommended for optimal analysis sensitivity and stringency.

Enrichment analyses were run using two different methods. First, an exploratory assessment, IPA Core Expression analysis was run using IPA microRNA target filter output. The IPA microRNA target filter queries the TargetScan, TarBase, and miRecords databases, which contain predicted and experimentally validated miRNA-mRNA interactions at all prediction confidence levels. Enriched Canonical Pathways and Diseases/Biological Functions were identified using an uncorrected significance threshold of 0.05. Full IPA analysis settings are available in Supplementary Materials (Supplementary Table 1). Second, a more stringent approach, the DIANA miRPath tool, v.3 (Vlachos et al., 2015) was used to identify enriched KEGG pathways from transcripts identified by the microT-CDS algorithm. miRPath was run using the “Pathways Union” algorithm, with “Unbiased Empirical Distribution” selected as the enrichment method. In contrast to the commonly used miRPath “Genes Union” setting, which is similar to the approach taken by the IPA Core analysis, Pathways Union utilizes an FDR corrected, merged *p*-value that represents the likelihood a certain biological pathway is targeted by at least one of the input miRNAs. Further, use of an empirical distribution, which includes all transcripts predicted to be targets of miRNAs (vs. the entire murine transcriptome), has been suggested to reduce algorithm bias and increase prediction accuracy (Bleazard et al., 2015).

### Statistical Analysis

Behavioral data were analyzed in SPSS (IBM, Armonk, NY, United States) with factorial analysis of variance (ANOVA). Independent factors included stress (Control vs. CVSS), strain (C57BL/6J vs. BALB/cJ), sex (male vs. female), day (1–11), concentration (morphine: 0.3/0.7/1 or quinine: 0.2/0.4/0.55 mg/ml), and drug group (chronic saline vs. chronic drug) where appropriate. Interactions that included multiple variables were interpreted with successive ANOVAs with fewer factors. Tukey’s *post hoc* test was used and the alpha level set at 0.05.

## Results

### Morphine Sensitization

**FIGURE 1:**
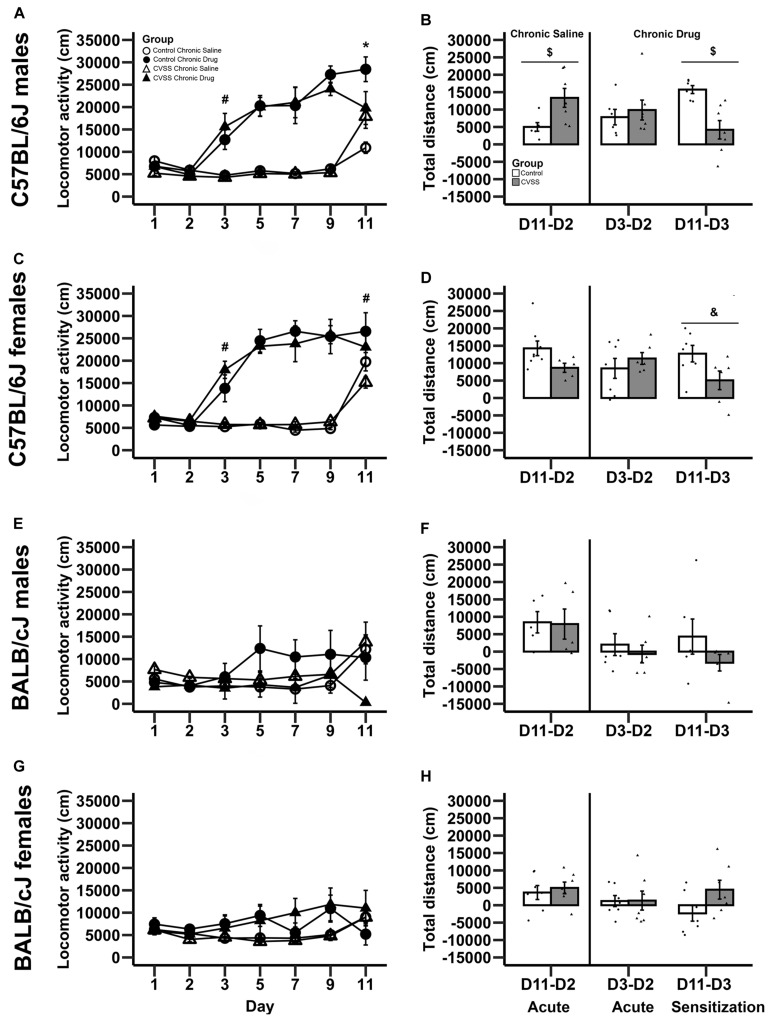
Morphine-induced acute behavioral stimulation and sensitization in adult C57BL/6J and BALB/cJ mice. Daily mean locomotor distance (cm) ±SEM are presented in panels: **(A)** C57BL/6J males, **(C)** C57BL/6J females, **(E)** BALB/cJ males, and **(G)** BALB/cJ females. Within-subject morphine stimulant and sensitization scores (mean ± SEM) are presented in panels: **(B)** C57BL/6J males, **(D)** C57BL/6J females, **(F)** BALB/cJ males, and **(H)** BALB/cJ females. Chronic saline animals are on the left side and chronic drug treated mice on the right side in each panel, with sensitization in the chronic drug treated mice shown in the far right-hand bars. *N* = 5–8 per stress and drug condition. *p < 0.05 for the stress × drug group interaction; #*p* < 0.05 for the main effect of drug group; $*p* < 0.05 for the main effect of stress; &*p* = 0.055 for the main effect of stress.

The impact of adolescent social stress on morphine sensitization varied by strain. Adult C57BL/6J mice exposed to adolescent social stress had blunted morphine sensitization, but BALB/cJ mice did not (Figures 1A–D vs. E–H). In the overall analysis with strain, stress, sex, drug group, and day as factors, we observed many significant effects involving strain ranging from a main effect of strain to 4-way interactions involving this factor, thus we conducted further analyses split by strain (see Supplementary Table 2 for full ANOVA results).

In C57BL/6J mice, we observed multiple significant effects (see Table 3 for full results) including a day × stress × sex × drug group interaction (*F*_6_,_264_ = 2.5, *p* < 0.05). To decompose this 4-way interaction, follow up analyses were conducted in each sex separately. In male C57BL/6J mice, we observed a significant main effect of day (*F*_6_,_132_ = 38.4, *p* < 0.001), drug group (*F*_1_,_22_ = 88.2, *p* < 0.001), day × drug group (*F*_6_,_132_ = 22.5, *p* < 0.001), and day × stress × drug group interactions (*F*_6_,_132_ = 4.3, *p* < 0.01). The interactions between day and group are consistent with the experimental design and suggest that the pattern of activity across days is dependent on the experimental drug group. Thus, further analyses were limited to the key days that examined between-groups acute locomotor stimulation (Day 3) and sensitization (Day 11) (Gubner et al., 2014; Miller and Kamens, in press). On Day 3, the first day of morphine exposure in the chronic drug group, C57BL/6J mice showed increased locomotion in response to morphine injection compared to the chronic saline group that received saline, indicating the expected morphine stimulant response (main effect of drug group: *F*_1_,_22_ = 24.4, *p* < 0.01). There were no other main effects or interactions with stress on Day 3. On Day 11, the 5th day of morphine treatment in the chronic drug group compared to the first day of morphine exposure in the chronic saline group, there was a significant main effect of drug group (*F*_1_,_22_ = 11.7, *p* < 0.01) and a stress × drug group interaction (*F*_1_,_22_ = 7.6, *p* < 0.05). *Post hoc* analyses demonstrated that for control animals there was a significant difference between the chronic drug and chronic saline groups (*p* < 0.05), indicating the expected morphine sensitization. In contrast, there was no significant difference between the chronic drug and chronic saline groups in the CVSS animals, suggesting attenuated morphine sensitization.

**TABLE 3:** Morphine Sensitization ANOVA Output from C57BL/6J mice including Day, Stress Condition, Sex, and Drug Group as independent factors.

Factor	Df	*F*	*p*-value
Day	6, 264	85.9	<0.001*
Day × Stress Condition	6, 264	1.3	0.3
Day × Sex	6, 264	0.7	0.7
Day × Drug Group	6, 264	55.1	<0.001*
Day × Stress Condition × Sex	6, 264	0.9	0.5
Day × Stress Condition × Drug Group	6, 264	2.6	0.02*
Day × Sex × Drug Group	6, 264	1.2	0.3
Day × Stress Condition × Sex × Drug Group	6, 264	2.5	0.02*
Stress Condition	1, 44	0.1	0.8
Sex	1, 44	2.0	0.2
Drug Group	1, 44	152.8	<0.001*
Stress Condition × Sex	1, 44	0.1	0.8
Stress Condition × Drug Group	1, 44	0.1	0.7
Sex × Drug Group	1, 44	0.2	0.6
Stress Condition × Sex × Drug Group	1, 44	0.2	0.7

*p < 0.05.

A similar pattern was observed in male C57BL/6J mice with the within-group analyses. In chronic saline exposed male C57BL/6J mice, CVSS males had a more robust acute locomotor response to morphine compared to unstressed control males (Day 11–Day 2; *t*_11_ = −2.6, *p* < 0.05; Figure 1B). But there was no significant difference in the acute locomotor response to morphine between stress and control animals in the chronic drug groups (Day 3–Day 2). Findings from the within-group measure of sensitization (Day 11–Day 3), replicated the between-group analysis (Day 11 above). Control male C57BL/6J mice exposed to chronic morphine exhibited robust behavioral sensitization that was attenuated in CVSS animals (*t*_11_ = 3.8, *p* < 0.01; Figure 1B).

In female C57BL/6J mice, we observed a significant main effect of day (*F*_6_,_132_ = 48.7, *p* < 0.001), group (*F*_1_,_22_ = 68.4, *p* < 0.001), and a day × drug group interaction (*F*_6_,_132_ = 34.3, *p* < 0.001). Importantly, in female C57BL/6J mice the main effect of stress and interactions with this variable were not significant. When examining days indicative of acute stimulation (Day 3) and sensitization (Day 11) there were significant main effects of drug group (*t*_24_ = −5.5, *p* < 0.001, *t*_24_ = −2.4, *p* < 0.05, respectively). Compared to chronic saline mice, chronic drug mice exhibited greater locomotor activity on Day 3 (mean ± SEM, 5541 ± 258, 15732 ± 1863, respectively), indicative of the acute stimulant response, and a greater response on Day 11, indicative of between-groups sensitization (mean ± SEM, 17994 ± 1463, 24929 ± 2475, respectively). In the within-group analysis, no significant stress group differences were observed for the acute locomotor response in either the chronic saline or chronic drug groups. When behavioral sensitization (Day 11 – Day 3) was examined, there was a statistical trend (*p* = 0.055) where female C57BL/6J mice exposed to chronic adolescent social stress had blunted morphine sensitization compared to control animals (Figure 1D).

In BALB/cJ mice, the 100 mg/kg dose of morphine did not result in locomotor stimulation or sensitization (Figures 1E–H). The overall analysis within this strain demonstrated a main effect of day (*F*_6_,_252_ = 4.9, *p* < 0.001) and a day × drug group interaction (*F*_6_,_252_ = 6.6, *p* < 0.001). *Post hoc* analysis found no significant effects on Day 3 or Day 11. Further, there were no significant effects observed in the within-group analysis for either acute stimulation or sensitization in BALB/cJ mice.

### Morphine Consumption

Five C57BL/6J mice (spread among stress conditions and sex) died during the consumption study. Veterinarian examination revealed that the animals did not have food in their stomachs suggesting drug-induced anorexia. Although we followed a published 24-h morphine access consumption paradigm (Eastwood and Phillips, 2014), future work should consider limiting drug availability to 18 h to decrease the chance of such an adverse effect (Kamens et al., 2005).

**FIGURE 2:**
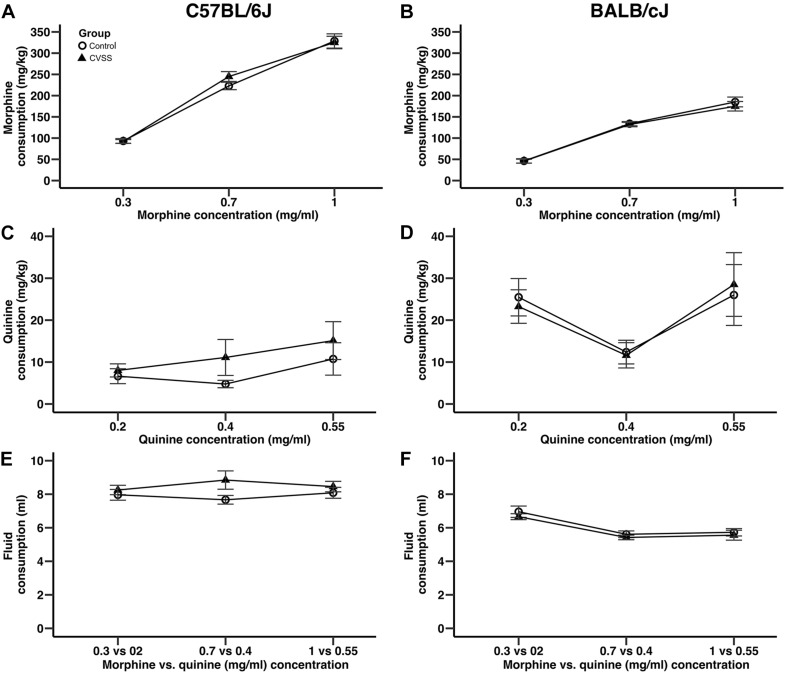
Adolescent stress did not influence morphine or quinine consumption in adult C57BL/6J or BALB/cJ mice. Morphine consumption and quinine consumption (mean ± SEM) in **(A,C)** C57BL/6J and **(B,D)** BALB/cJ mice. Mean (±SEM) total fluid intake in **(E)** C57BL/6J and **(F)** BALB/cJ mice. *N* = 22–28 per strain and stress condition.

Adolescent social stress did not influence adult morphine consumption in either C57BL/6J or BALB/cJ (Figure 2) mice. In an overall analysis of morphine consumption (mg/kg) there was a significant main effect of strain (*F*_1_,_95_ = 180.9, *p* < 0.001) and a strain × concentration interaction (*F*_2_,_190_ = 43.5, *p* < 0.001), thus C57BL/6J and BALB/cJ mice were separated for further analyses. In C57BL/6J mice, morphine consumption increased in a concentration dependent manner (Figure 2A; main effect of concentration: *F*_2_,_90_ = 348.8, *p* < 0.001, all *post hoc p* < 0.001), but no other significant main effects or interactions were observed. There were no significant effects on quinine consumption (Figure 2C). For total fluid intake, while there was a significant sex × concentration interaction (*F*_2_,_90_ = 3.3, *p* < 0.05), *post hoc* comparison suggest this was driven by a trend (*p* = 0.06) for the males to consume more fluid compared to females (8.9 ± 0.6, 7.7 ± 0.3, respectively), but only when the middle morphine concentration (0.7 mg/ml) was available.

Similar to the results observed in C57BL/6J mice, in BALB/cJ mice drug concentration influenced intake, but no main effects or interactions with stress condition were observed. BALB/cJ mice consumed significantly more morphine when the 0.7 and 1 mg/ml morphine concentrations were available compared to the 0.3 mg/ml concentration (Figure 2B, main effect of concentration: *F*_2_,_100_ = 228.4, *p* < 0.001, all *post hoc p* < 0.001). Importantly, there were no main effects or interactions with stress condition. In BALB/cJ mice, there was a significant main effect of concentration on quinine consumption (*F*_2_,_100_ = 6.0, *p* < 0.01). Here, quinine consumption was lower when the middle quinine concentration was available relative to the low or high concentrations (Figure 2D, all *post hoc p* < 0.05). Finally, when total fluid intake was examined there was a significant main effect of concentration (*F*_2_,_100_ = 27.2, *p* < 0.001) and sex (*F*_1_,_50_ = 4.9, *p* < 0.05). BALB/cJ mice drank significantly more fluid when the 0.3 mg/ml (6.8 ± 0.2) morphine concentration was available relative to both the 0.7 (5.5 ± 0.1) or 1 (5.6 ± 0.2) mg/ml concentrations (Figure 2F, all *post hoc p* < 0.001). Overall, male BALB/cJ mice consumed more fluid than female BALB/cJ mice (6.2 ± 0.1, 5.7 ± 0.2, respectively).

### Acute Stress

**FIGURE 3:**
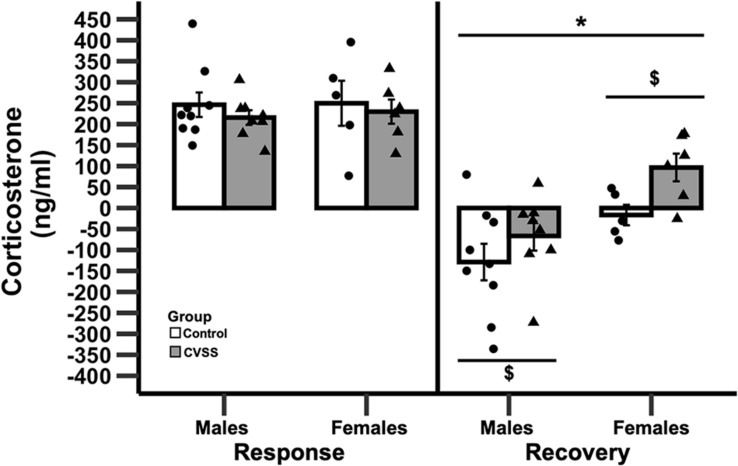
Change in corticosterone following 15 min of restraint stress in adult C57BL/6J mice. Mean corticosterone (ng/ml) ±SEM in C57BL/6J male and female mice. *N* = 14 per stress condition. *p < 0.05 for the main effect of sex; $*p* < 0.05 for the main effect of stress group.

Adult C57BL/6J mice exposed to adolescent social stress had a slower recovery of CORT levels following acute restraint stress compared to control mice (Figure 3). Baseline levels of CORT were not influenced by stress or sex (data not shown). We then examined stress reactivity (30 min CORT–baseline CORT) and again no significant effects were observed. In contrast, when recovery (90 min CORT–30 min CORT) was examined there was a significant main effect of stress (*F*_1_,_24_ = 12.1, *p* < 0.01) and sex (*F*_1_,_24_ = 4.9, *p* < 0.05), but the interaction of these variables was not significant. Mice exposed to CVSS had a slower CORT recovery compared to control animals (3.3 ± 32.5, −88.7 ± 32.2, respectively) and females recovered more slowly than males (45.1 ± 26.9, −99.5 ± 28.5, respectively).

### Prefrontal Cortex (PFC) miRNA Gene Expression

**FIGURE 4:**
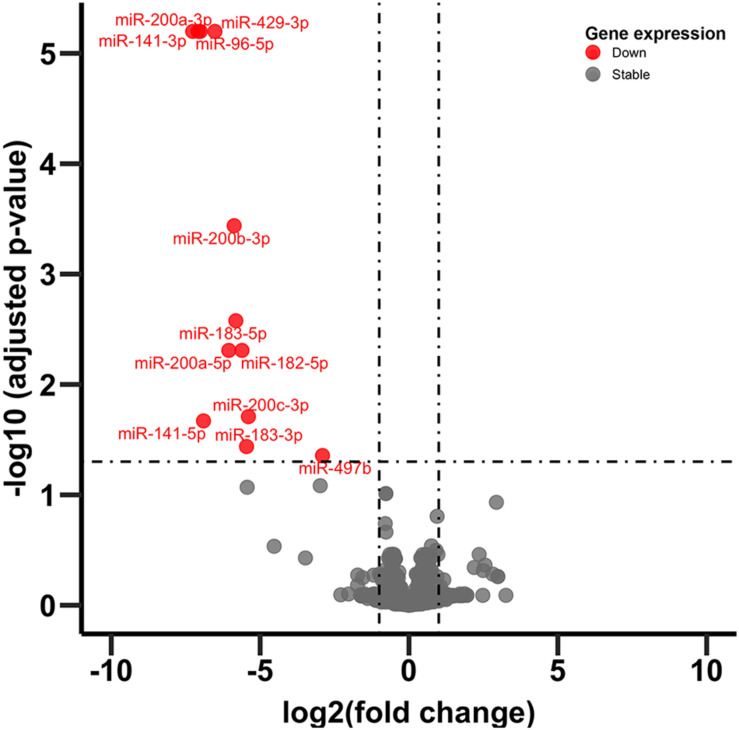
Volcano plot of miRNA expression. Dots in red indicate miRNA significantly downregulated following CVSS compared to control (FDR < 0.05).

In adult C57BL/6J male mice, of the 904 miRNA identified through RNA sequencing, twelve (miR-429-3p, miR-200a-3p, miR-96-5p, miR-141-3p, miR-200b-3p, miR-183-5p, miR-200a-5p, miR-182-5p, miR-200c-3p, miR-141-5p, miR-183-3p, and miR497b; Figure 4) were downregulated following adolescent social stress (for full results see Supplementary Table 3). The DIANA microT-CDS algorithm, run with the 12 differentially expressed miRNA, predicted 5433 total miRNA-mRNA relationships, with 2820 unique mRNA targets. Five significant KEGG pathways were identified by miRPath: MAPK signaling, Glycosphingolipid biosynthesis (lacto and neolacto series), AMPK signaling, Glycosphingolipid biosynthesis (globo series), and Gap junction (see also Table 4). At a significance threshold of 0.05, IPA Core Expression analysis identified 500 enriched Diseases/Biological Functions and 267 enriched Canonical Pathways. See Table 5 for the top five enrichment terms in each category and Supplementary Tables 4,5 for full IPA output.

**TABLE 4:** miRPath output.

KEGG pathway	*p*-value	#genes	#miRNAs
MAPK signaling pathway	0.015	5	1
Glycosphingolipid biosynthesis–lacto and neolacto series	0.018	2	1
AMPK signaling pathway	0.027	4	1
Glycosphingolipid biosynthesis–globo series	0.027	3	2
Gap junction	0.048	2	1

**TABLE 5:** IPA output.

IPA top five canonical pathways	*p*-value	
Molecular mechanisms of cancer	3.98E-11	
BMP signaling pathway	1E-10	
Synaptogenesis signaling pathway	2.75E-10	
Cardiac hypertrophy signaling	3.24E-09	
GNRH signaling	1.66E-08	

**IPA disease/function annotation**	***p*-value**	**#molecules**

Non-hematological solid tumor	7.44E-208	4133
Non-hematologic malignant neoplasm	1.25E-204	4118
Cancer	3.74E-190	4164
Malignant solid tumor	1.4E-189	4151
Non-melanoma solid tumor	5.4E-189	4079

## Discussion

In the present study we examined the effect of adolescent social stress on adult morphine behaviors in two inbred strains of mice, and characterized two key biological mechanisms that may account for decreased morphine sensitization in C57BL/6J mice exposed to adolescent social stress. We found that adolescent social stress blunted morphine sensitization in adult C57BL/6J, but not BALB/cJ, mice, an effect that was more robust in males. This behavioral effect of stress emerged after repeated drug administration, as there was little evidence of a change in the acute locomotor response to morphine. Adolescent stress did not influence adult voluntary morphine consumption in either strain. We observed long-term changes in both the recovery of the CORT response following an acute stressor and in the expression of adult miRNA in the PFC. It is possible that these changes reflect mechanisms linked to the altered morphine response. These data indicate that a gene-environment interaction may influence opioid behaviors.

Our results indicate that male C57BL/6J mice are particularly vulnerable to lasting effects of adolescent stress. In both the within- and between-groups analysis we observed that control animals developed normal morphine-induced behavioral sensitization, but this was blunted in animals that were exposed to social stress in adolescence. A similar statistical trend (*p* = 0.055) was observed in female C57BL/6J mice in the within-group analysis. Due to the complex design of this experiment, there were 5–8 mice per sex, stress condition, and drug group; thus, it is possible we were underpowered to detect significant effects. The fact that there is a known statistical power advantage of within-subject designs may explain why we observed a trend in the female C57BL/6J mice using this analysis (Keppel, 1991). Our findings are consistent with the work of Shaham et al. (1995) in adult male rats. Here the authors showed that restraint stress not paired with an intra-VTA morphine injection exhibited less locomotor sensitization compared to morphine only treated rats or animals who had 15 min of restrain stress paired with a morphine injection. Interestingly, this pattern only emerged with an intra-VTA morphine injection and not when the drug was administered systemically.

Our findings that adolescent stress blunts morphine sensitization aligns with two other pre-clinical studies. In work by Coudereau, no morphine conditioned place preference (8, 16, 64, or 100 mg/kg) was observed in male NMRI mice that were exposed to 30 days of social isolation starting in adolescence (Coudereau et al., 1997). Similar abolishment of morphine conditioned place preference was observed in male Lister hooded rats that were isolated for 6 weeks starting at weaning (Wongwitdecha and Marsden, 1996). Both of these studies incorporated isolation stress similar to ours, but it is possible that these effects extent to other stressors as well. One limitation of the current work is that we utilized only a single high dose of morphine, thus we can’t determine if adolescent stress shifts sensitivity to morphine. A similar series of experiments with additional morphine doses may provide some insight, but when our results are compared to earlier findings with morphine conditioned place preference (Wongwitdecha and Marsden, 1996; Coudereau et al., 1997) it could be hypothesized that adolescent stress blocks normal behavioral responses to opioids. Obviously, additional work is necessary to confirm this hypothesis.

Clinical literature reports psychological stress as a risk factor for initiation of opioid use (Hassanbeigi et al., 2013). It is interesting that in the pre-clinical literature, adolescent stress appears to dampen sensitivity to opioids. This has now been replicated using both morphine sensitization in the current study and the rewarding effects of morphine in prior studies (Wongwitdecha and Marsden, 1996; Coudereau et al., 1997). How to reconcile these data is not yet apparent. Human studies suggest that individuals who use opioids tend to experience stressful situations but they also use multiple drugs in addition to opioids, many have psychiatric diseases, and genetic factors increase risk among some individuals. In animal models, these external factors are controlled. It is possible that the decreased sensitivity to opioids observed in pre-clinical models is reflected in humans as individuals seeking out more drug to feel the same effect. Alternatively, it remains possible that pre-clinical researchers have not examined the right drug phenotype and/or this stressor doesn’t mimic the experience of adolescent stress in humans. For example, there are no studies to date that have examined the effect of adolescent stress on animal models of intravenous drug self-administration, a gold standard model of drug reinforcement.

In humans, psychological distress has been reported to be a greater risk factor for opioid abuse in females compared to males (Jamison et al., 2010). In our data, we report that our adolescent stressor appears to result in stronger effects on morphine sensitization in male mice. As discussed above, we did see a trend in female mice and we may have observed stronger effects with an increased sample size. It is difficult to compare these sex differences with other pre-clinical research because female animals have not been included in many studies. Specifically, the two studies that have examined effects of adolescent stress on opioid conditioned place preference only used male rodents (Wongwitdecha and Marsden, 1996; Coudereau et al., 1997). Clearly this is an area that requires further investigation.

The differences in morphine behavioral responses appear to emerge after repeated drug exposure as we saw little indication that stress altered the acute locomotor response to morphine. We observed that CVSS increased morphine stimulation in the male C57BL/6J chronic saline group, but not in the chronic drug group or with the between-groups analysis. These findings are consistent with a previous study that demonstrated that 14 days of restraint stress in adult C57BL/6J mice had no effect on acute morphine locomotor stimulation (Xu et al., 2014). However, these results differ from prior research in adult rats that have demonstrated that stress (restraint, handling, social defeat, foot shock, or social isolation) potentiates morphine-induced locomotor stimulation (Leyton and Stewart, 1990; Deroche et al., 1994; Stöhr et al., 1999). The stressors in these studies varied in duration and number of exposures. Stöhr et al. (1999) demonstrated that one 60-min restraint stress was not enough to potentiate morphine locomotor stimulation, but three exposures to restraint stress, handling or social defeat did. The stressors in the other papers were longer with five sessions of 20 min foot shock or 17 days of continuous social isolation in adulthood. These procedural differences make it difficult to draw strong conclusions, but it is interesting to note that while significant effects of stress exposure on morphine stimulation have been observed in rats, there is currently little evidence in C57BL/6J mice.

In the present work, we observed no difference in morphine consumption following adolescent social stress. Our findings align with work in adult male C57BL/6J mice. Exposure to chronic social stress increased morphine preference immediately (1 day) after stress exposure, but the effect was no longer present when tested 14 days post-stress exposure (Cooper et al., 2017). In the current experiment, mice were given access to morphine beginning 8–11 days following the stress exposure. We originally hypothesized that stress during adolescence would result in long-lasting differences in drug consumption because of the critical time in brain development, but that was not observed with morphine consumption. Instead, the effects of stress on opioid consumption may be time-dependent and only evident immediately after stress exposure. This is important when considering factors that could lead to drug use initiation.

In both the morphine sensitization and consumption experiments, we found pronounced differences between C57BL/6J and BALB/cJ mice. These differences confirm prior research demonstrating strain differences in morphine behaviors (Belknap et al., 1993; Semenova et al., 1995; Kest et al., 2002; Kennedy et al., 2011). Specifically, these results align with literature showing that C57BL/6J mice consume significantly more morphine than BALB/cJ mice (Belknap et al., 1993). In the current study our paradigm produced robust behavioral sensitization in C57BL/6J mice, but not in BALB/cJ mice. To date there is little published data on morphine sensitization in BALB/cJ mice. In studies examining acute locomotor activation in adult C57BL/6J and BALB/cJ mice, these strains exhibited similar stimulation in response to 32 mg/kg morphine (Belknap et al., 1998). It is possible that the dose of morphine (100 mg/kg) we chose was too high to produce sensitization in the BALB/cJ strain. Thus, while we can say CVSS did not influenced morphine consumption in either C57BL/6J or BALB/cJ mice, our conclusion on morphine sensitization is limited to the specific dose tested in this experiment. It is critical to recognize that different effects may be observed on other morphine behaviors or in response to other doses of morphine used in our paradigms.

Here we show that exposure to chronic social stress in adolescence changes the physiological response to an acute stressor in adulthood. In particular, we found that it took stressed mice longer to recover following a 15 min restraint stress. This finding is in line with research suggesting that animals chronically exposed to one type of stressor have an altered hormonal response to a novel stressor (Romeo, 2013). For example, adolescent rats exposed to 7 days of a cold room stressor then restraint stress had a delayed recovery of CORT to the restraint stress. In contrast, this effect was not observed in adult rats or in adolescent rats exposed to 8 days of restrain stress (Lui et al., 2012). In this prior study, all stress exposures occurred during adolescence, but the current findings suggest that this altered physiological response to an acute stressor may last into adulthood.

The HPA system appears to be involved in opioid responses. In adult rats, repeated exposure to restraint stress increased the locomotor response to an acute injection of morphine, an effect which was showed to be dependent on stress-induced corticosterone secretion (Deroche et al., 1992). Additionally, short term isolation of adult rats produced a similar increase in morphine-induced locomotor activity; a response which again was blocked by removal of the adrenal glands resulting in no stress-induced corticosterone secretions (Deroche et al., 1994). In both of these cases the outcome was locomotor activity following a single injection of morphine. To our knowledge little data exist on the role of the HPA system in sensitization observed following chronic morphine administration.

In general, the PFC is thought to activate the HPA axis, although the specific mechanism depends on the stimulus (Cerqueira et al., 2008). Further, this connection is weakened by chronic stress (Sullivan and Gratton, 1999). Here we found no difference in the CORT response to an acute stress, but instead found a delayed recovery to baseline. We have found no other studies that report similar findings; thus, replication of this work will be important.

In this study we observed 12 miRNA in the adult PFC that were downregulated following adolescent social stress. We chose to examine differential gene expression in the PFC because of our prior work which demonstrated lasting changes in neuronal excitability in this region (Caruso et al., 2018a). A differentially expressed miRNA of interest that we identified was miR-183. This finding is consistent with a prior study that showed chronic immobilization stress in adulthood decreased the expression of this miRNA in the amygdala and CA1 region of the hippocampus (Meerson et al., 2010). Interestingly, in that study an upregulation was identified after acute immobilization stress, suggesting that the duration of stress exposure is important. Time-dependent effects of stress exposure on other miRNAs have also been observed. For example, miR-200a-3p was shown to be down regulated in the PFC of rats exposed to 4 weeks of chronic unpredictable stress in adulthood; a finding similar to our own. In contrast, after 12 weeks of stress exposure this same miRNA was upregulated (Satyanarayanan et al., 2019). We believe that this investigation produced the first known data to examine the global patterns of PFC miRNA expression following adolescent stress. Given that these small RNA molecules regulate mRNA expression, our findings could contribute to the altered behavioral responses we observed, but additional work is necessary to confirm this hypothesis. Further, these data provide additional support that repeated exposure to stress in adolescence may compromise typical development of the PFC.

To our knowledge none of the miRNA we found differentially expressed have been linked to addiction phenotypes. miRNA have emerged in the last decade as a potential regulator of addiction (for review see Smith and Kenny, 2018). In this work, changes in miRNA are thought to be an epigenetic mediator of mRNA gene expression and a mechanism for altering brain plasticity in response to drug exposure. Thus, researchers have looked at how miRNA are changed in response to drugs. One example from clinical literature is that specific miRNA are differentially expressed in patients with opioid use disorder compared to healthy controls (Hsu et al., 2019). Here we suggest that exposure to stress in adolescence (in the absence of any drug exposure) also changes miRNA expression. Thus, these stress-induced alterations in miRNA levels may predispose individuals to being more/less sensitive to drug exposure and possibly alter later drug use.

Functional enrichment analysis of the differentially expressed miRNA and their predicted mRNA targets identified proCancer-related pathways among the top enrichment terms in both miRPath and IPA analyses (Supplementary Tables 4, 5). Cancer-related signaling tends to involve ubiquitous molecules and pathways that often also participate in neurological functioning. For example, the top enriched miRPath KEGG pathway was MAPK signaling (Table 4), a biological pathway well known to mediate cancer formation as well as learning and memory (Atkins et al., 1998; Wagner and Nebreda, 2009). Further, in addition to their known role in tumor formation (Inokuchi et al., 1987), glycosphingolipids have recently been implicated in addiction (Kalinichenko et al., 2018). IPA core analysis identified overall top enrichment terms related to cancer and synaptogenesis (Supplementary Table 5). It is also notable that the top IPA Diseases/Functions terms under the subcategory “Physiological System Development and Function” was “Nervous System Development and Function.” Thus, adolescent stress appears to alter expression of adult miRNAs broadly regulating signaling pathways related to cancer and nervous system development/functioning. These specific miRNA and pathways serve as mechanisms by which adolescent stress my change later opioid behaviors.

The current work demonstrates a gene-environment interaction that predisposes certain individuals to opioid behaviors following adolescent stress. Specifically, adult C57BL/6J mice exposed to adolescent social stress had attenuated morphine sensitization compared to control animals, whereas BALB/cJ mice did not. It remains possible that adolescent stress may impact morphine sensitization in BALB/cJ if a more optimal dose of this drug was used in this strain. This is a critical point; our conclusion of a gene-environment interaction is limited to the two strains we tested and to sensitization to a 100 mg/kg dose of morphine. Interestingly, this is not the first time we observed a strain × stress dependent effects with this stressor; a similar finding was seen with nicotine behaviors, with altered sensitivity to nicotine in male BALB/cJ, but not C57BL/6J mice (Caruso et al., 2018a,b). Given that the majority of addiction research utilizes C57BL/6J mice it is important to recognize that we may miss gene-environment interactions if utilizing only a single mouse strain. This limitation extends to our own work where we conducted our physiological assays in only C57BL/6J mice. While we performed follow-up work on C57BL/6J animals because of the behavioral differences we observed, if we had included both strains in our physiological measurements, we may have observed gene-environment interactions with those phenotypes as well.

## Data Availability Statement

Sequencing data generated for this study can be found in GEO accession number GSE172077.

## Ethics Statement

This study was reviewed and approved by The Pennsylvania State University Institutional Animal Care and Use Committee.

## Author Contributions

HK, CM, JC, and SC designed the study. HK, CM, and JC performed the experiments. HK, CM, JC, DZ, WH, CS, AS, IA, and SC analyzed the data. HK, WH, TG, DF, PG, and SC provided conceptual input into the project and edited the manuscript. HK, JC, and DZ wrote the manuscript. All authors contributed to the article and approved the submitted version.

## Conflict of Interest

The authors declare that the research was conducted in the absence of any commercial or financial relationships that could be construed as a potential conflict of interest.

## Footnotes

1 http://diana.imis.athena-innovation.gr/DianaTools/
